# Stochastic Modeling of Smartphones GNSS Observations Using LS-VCE and Application to Samsung S20

**DOI:** 10.3390/s23073478

**Published:** 2023-03-26

**Authors:** Farzaneh Zangenehnejad, Yang Gao

**Affiliations:** Department of Geomatics Engineering, Schulich School of Engineering, University of Calgary, Calgary, AB T2N 1N4, Canada

**Keywords:** smartphone positioning, stochastic model, least square variance component estimation (LS-VCE), precise point positioning (PPP)

## Abstract

In recent years, numerous smartphones have been equipped with global navigation satellite system (GNSS) technology, enabling individuals to utilize their own devices for positioning and navigation purposes. In 2016, with the launch of a mobile app by Google, namely GnssLogger, smartphone users with Android version 7 or higher were able to record raw GNSS measurements (i.e., pseudorange, carrier phase, Doppler, and carrier-to-noise density ratio (C/N0)). Since then, enhancing the accuracy and efficiency of smartphone positioning has become an interesting area of research. Precise point positioning (PPP) is a powerful method providing precise real-time positioning of a single receiver, and it can be applied to smartphone observations as well. Achieving high-precision PPP requires selecting appropriate functional and stochastic models. In this study, we investigate the development of more reliable stochastic models for smartphone GNSS observations. The least-square variance component estimation (LS-VCE) method is applied to double-difference (DD) pseudorange and carrier phase observations from two Samsung S20s to obtain appropriate variances for GPS and GLONASS. According to the results, there is no significant correlation between the pseudorange and carrier phase observations of GPS and GLONASS on the L1 frequency. Furthermore, the quality of GLONASS carrier phase observations is comparable to that of GPS. The model’s performance is then assessed with respect to single-frequency precise point positioning (SF-PPP) using a dataset collected in kinematic mode from a Samsung S20 smartphone. A significant improvement of 25.1% and 32.7% on the root-mean-square (RMS) of horizontal positioning and the 50th percentile error, respectively, was achieved when employing the obtained stochastic model.

## 1. Introduction

The widespread demand for precise position information in mass-market applications has led to a significant increase in the use of low-cost and ultra-low-cost receivers. In recent years, many smartphones have also been equipped with advanced carrier phase tracking measurement technology to support precise position determination. During the “Google I/O” conference in May 2016, Google announced that the raw GNSS measurements, such as pseudorange, carrier phase, Doppler shift, and carrier-to-noise density ratio (C/N0) observations, would be available on Android Nougat (version 7) or higher operating systems. With the availability of several hundred smartphone models in the market that can provide raw GNSS observations, there is an increasing demand for research on improving smartphone positioning performance [1,2].

While the GNSS signals can be effectively used for outdoor localization purposes, signal attenuation or blockage prevents their employment in indoor settings. As a result, various alternative technologies, including Frequency modulation (FM), Ultra-wideband (UWB), Radio Frequency Identification (RFID), Wi-Fi, Bluetooth, and cellular networks as well as inertial sensors, are being utilized to meet the growing demand for indoor positioning [3,4,5,6,7,8]. In this contribution, however, we focus on the application of the GNSS technique for outdoor positioning.

The precise point positioning (PPP) method is a single-receiver global navigation satellite system (GNSS)-based precise positioning technique. It is different from the real-time kinematic (RTK) technique, which typically requires a base station [9,10]. Currently, there is a high demand for improving PPP accuracy with smartphones, especially in kinematic mode. Since the quality of smartphone observations is not as good as that of high-end geodetic receivers, there are several issues that must be addressed. Choosing appropriate stochastic models is crucial for achieving high-precision positioning where the stochastic model describes the statistical properties of observables. The elevation-dependent weighting functions are generally employed to build a weighting model for the high-end geodetic receivers’ observations. However, the elevation angle is not the only parameter that can be used to create a stochastic model for smartphone observations. The C/N0 value can also be effectively used to assign proper weights to the smartphone GNSS observations based on signal strength. The C/N0 refers to the ratio of the carrier power and the noise power per unit bandwidth [11]. It can therefore be considered a powerful indicator of the GNSS signal strength in the sense that a larger C/N0 indicates a stronger signal while a lower C/N0 shows a weaker signal. Recent studies have shown that the C/N0-dependent weighting method is superior to the elevation-dependent weighting method when using the GNSS observations obtained from Android smartphones. We will mention a few of these studies in the following.

Liu et al. [12] investigated the quality of raw GNSS observations of smartphones in terms of the C/N0, noise, tracking capability of the carrier phase, and velocity estimation. They found that there is a stronger correlation between the pseudorange accuracy and C/N0 rather than the satellite elevation angle. Therefore, using an elevation-dependent weighting model is not suitable for low-cost receivers, and a C/N0 weighting model is a better choice. Banville et al. [13] also proposed a C/N0 weighting model as an alternative to an elevation-dependent weighting model based on factors such as multipath noise, chipping length, or wavelength and C/N0. This weighting model was later utilized by Shinghal and Bisnath [14]. Employing the C/N0 weighting model instead of an elevation-dependent weighting model has been also suggested in other studies [15,16,17]. Wang et al. [18] developed the Smart-PPP software application for smartphones, which achieved sub-meter float positioning accuracy in horizontal directions by utilizing a C/N0-based weighting model. The weighting model was based on an exponential function that took into account various factors such as the instantaneous C/N0 of the observations, a maxi-mum C/N0 value for cutoff, and the corresponding pseudorange noise at maximum C/N0. Further details on this weighting model are available in their study [18]. Li et al. [19] also proposed a combined elevation angle and C/N0 weighting method for smartphone-based GNSS PPP by normalizing the C/N0-derived variances to the scale of the elevation-angle-derived variances. Their findings showed that this method improved the three-dimensional positioning accuracy by 22.7% and 24.2% in an open-sky area, and by 52.0% and 26.0% in a constrained visibility area, compared with the elevation-angle-only and C/N0-only weighting methods, respectively. Miao et al. [20] also proposed a new weighting model that considers the variation range of C/N0, providing a better weighting model than the traditional weighting model, thus improving the positioning accuracy. The results indicated that the L5/E5a/B2a signals could generally obtain higher IAR fix-rate and positioning accuracies than the L1/E1/B1 signals.

To date, the existing stochastic models are mainly either elevation-dependent or C/N0-based weighting models. Our contribution proposes a more reliable stochastic model that combines both elevation-dependent and C/N0 weighting methods. This approach takes into account the impact of both elevation angles and C/N0 records on different GNSS constellations and observation types (i.e., pseudorange and carrier phase observations) to adjust the stochastic model accordingly. The parameters in such a stochastic model can be estimated by using the least-squares variance component estimation (LS-VCE) method. The LS-VCE method is a well-established method for the estimation of unknown (co)variance components [21,22,23,24]. The method has been successfully employed to assess the noise characteristics of the high-end geodetic receivers [25,26,27]. In Zangenehnejad and Gao [28], the LS-VCE method was also applied to stochastic modeling of noisy smartphone GNSS observations but the effect of the elevation angle and C/N0 on different GNSS constellations and different observation types was not investigated. In this study, the weighting coefficients will be estimated separately for each constellation and each observation type. We will also estimate any possible correlation between the GNSS pseudorange and carrier phase observables.

This paper is organized as follows: The LS-VCE method is briefly reviewed in Section 2. How to apply the LS-VCE method to noise assessment of GNSS smartphone observations is also explained in this section. The reliable stochastic model will then be applied to the single-frequency PPP (SF-PPP) model in Section 3. In Section 4, the performance of the SF-PPP model applying the obtained reliable stochastic model is investigated using the GNSS observations from the Samsung S20 device in a kinematic mode. Finally, some conclusions are made in Section 5.

## 2. Stochastic Modeling of Smartphone Observations Using Least-Square Variance Component Estimation

### 2.1. Least-Squares Variance Component Estimation (LS-VCE)

Let us assume the linear(ized) model of observation equations as

(1) $$E\left(y\right)=Ax;D\left(y\right)=Q_{y}=\sum_{k=1}^{p}{\sigma_{k}Q}_{k}$$

where E and D are the expectation and dispersion operators, respectively, y is the *m*-vector of observations, x is the *n*-vector of the unknown parameters, A is the m×n design matrix, and $Q_{y}$ is the m×m covariance matrix of the observables. The structure of the covariance matrix $Q_{y}$ is expressed as an unknown linear combination of some known cofactor matrices. The estimation of these unknown (co)variance parameters $\sigma_{k};k=1,2,\ldots ,p$ is referred to as variance component estimation (VCE). There exist many VCE methods and among them we will make use of the least-square variance component estimation (LS-VCE). The unknown (co)variance components are estimated as $\hat{\sigma }={\left[\begin{array}{ccc} {\hat{\sigma }}_{1} & \ldots & {\hat{\sigma }}_{p} \end{array}\right]}^{T}=N^{-1}l$, in which the component of the matrix $N_{p\times p}$ and vector $l_{p\times 1}$ are as follows [24]:

(2) $$n_{ij}=\frac{1}{2}trace\left(Q_{i}Q_{y}^{-1}P_{A}^{⊥}Q_{j}Q_{y}^{-1}P_{A}^{⊥}\right)$$

and

(3) $$l_{i}=\frac{1}{2}trace\left({\hat{e}}^{T}Q_{y}^{-1}Q_{i}Q_{y}^{-1}\hat{e}\right)$$

where trace is the sum of the diagonal elements of a matrix, $P_{A}^{⊥}=I_{m}-A(A^{T}Q_{y}^{-1}A{)}^{-1}A^{T}Q_{y}^{-1}$ is an orthogonal projector, $I_{m}$ is an *m*-by-*m* identity matrix, and $\hat{e}=P_{A}^{⊥}y$ denotes the *m*-vector of residuals. For more details about the LS-VCE method, the reader may refer to [21,22,23,24].

### 2.2. Application of LS-VCE Method to the Noise Assessment of the Smartphone Observations

To assess the noise characteristics of the GNSS smartphone observables, the LS-VCE method can be applied to the double-differenced (DD) observations of two smartphones. For zero and short baselines (up to 10 m as used in this contribution), the DD atmospheric (ionospheric and tropospheric) delays can be ignored. In this case, the geometry-based model is of the form [29]

(4) $$\begin{array}{l} E\left({\Delta \nabla \Phi }_{a,b,j}^{s,k}\right)={\Delta \nabla \rho }_{a,b}^{s,k}+\lambda_{j}{\Delta \nabla N}_{a,b,j}^{s,k}\\ E\left({\Delta \nabla P}_{a,b,j}^{s,k}\right)={\Delta \nabla \rho }_{a,b}^{s,k} \end{array}$$

where Δ∇ represent the DD operator, *a* and *b* represent the base and rover stations, respectively; *s* and *k* denote the satellites, P and Φ stands for the pseudorange (code) and carrier phase observations in meter, respectively; ${\Delta \nabla \rho }_{a,b}^{s,k}$ is the DD receiver-satellite range, $\lambda_{j}$ is the corresponding carrier phase wavelength (m) on the *j*th frequency and ${\Delta \nabla N}_{a,b,j}^{s,k}$ is the DD ambiguity. The unknowns are the baseline components between the reference and rover receivers as well as the ambiguous term ${\Delta \nabla N}_{a,b,j}^{s,k}$.

In this contribution, we focus on the noise assessment of GNSS observations from the Samsung S20 device. Samsung S20 is a dual-frequency smartphone supporting L5/E5a frequencies for GPS and Galileo, respectively. To collect data for this study, we placed two Samsung S20 devices on top of a geodetic pillar with known coordinates, resulting in a very short baseline of approximately 5 cm. However, for some reason, they could not log Galileo observations as well as E1 and E5a frequencies. In addition, the number of GPS satellites with L5 observations is only one or two during the whole time of data collection. Therefore, in this contribution, we will employ the single-frequency GPS and GLONASS observations (*j* = 1). In this case, the unknowns are ${\Delta \nabla N}_{a,b,1}^{s,k,GPS}$ and ${\Delta \nabla N}_{a,b,1}^{s,k,GLO}$ (considering the known coordinates of the geodetic pillar).

Following [25,26], when dealing with DD observation types of one epoch, the structure of the stochastic model is as follows:

(5) $$Q_{y}^{DD}=blkdiag(Q_{GPS}^{DD},Q_{GLO}^{DD})$$

in which blkdiag constructs a block diagonal matrix from input arguments and $Q_{GPS}^{DD}$ and $Q_{GLO}^{DD}$ are the two covariance matrices of the DD GPS and GLONASS observations, respectively. Assuming $n_{1}$ and $n_{2}$ visible satellites for GPS and GLONASS, respectively, $Q_{GPS}^{DD}$ and $Q_{GLO}^{DD}$ can be expressed as follows:

(6) $$Q_{GPS}^{DD}=\left[\begin{array}{cc} a_{code}^{GPS} & \rho^{GPS}\\ \rho^{GPS} & a_{phase}^{GPS} \end{array}\right]⊗2\left[\begin{array}{cccc} \sigma_{{\left[1\right]}^{G}}^{2}+\sigma_{{\left[2\right]}^{G}}^{2} & \sigma_{{\left[1\right]}^{G}}^{2} & \cdots & \sigma_{{\left[1\right]}^{G}}^{2}\\ \sigma_{{\left[1\right]}^{G}}^{2} & \sigma_{{\left[1\right]}^{G}}^{2}+\sigma_{{\left[3\right]}^{G}}^{2} & \cdots & \sigma_{{\left[1\right]}^{G}}^{2}\\ \vdots & \vdots & \ddots & \vdots \\ \sigma_{{\left[1\right]}^{G}}^{2} & \sigma_{{\left[1\right]}^{G}}^{2} & \cdots & \sigma_{{\left[1\right]}^{G}}^{2}+\sigma_{{\left[n_{1}\right]}^{G}}^{2} \end{array}\right]$$

and

(7) $$Q_{GLO}^{DD}=\left[\begin{array}{cc} a_{code}^{GLO} & \rho^{GLO}\\ \rho^{GLO} & a_{phase}^{GLO} \end{array}\right]⊗2\left[\begin{array}{cccc} \sigma_{{\left[1\right]}^{R}}^{2}+\sigma_{{\left[2\right]}^{R}}^{2} & \sigma_{{\left[1\right]}^{R}}^{2} & \cdots & \sigma_{{\left[1\right]}^{R}}^{2}\\ \sigma_{{\left[1\right]}^{R}}^{2} & \sigma_{{\left[1\right]}^{R}}^{2}+\sigma_{{\left[3\right]}^{R}}^{2} & \cdots & \sigma_{{\left[1\right]}^{R}}^{2}\\ \vdots & \vdots & \ddots & \vdots \\ \sigma_{{\left[1\right]}^{R}}^{2} & \sigma_{{\left[1\right]}^{R}}^{2} & \cdots & \sigma_{{\left[1\right]}^{R}}^{2}+\sigma_{{\left[n_{2}\right]}^{G}}^{2} \end{array}\right]$$

in which ⊗ denotes the Kronecker product, the satellite number ${\left[1\right]}^{G}$ and ${\left[1\right]}^{R}$ are assumed to be the reference satellites for GPS (G) and GLONASS (R) constellations, respectively; $\rho^{GPS}$ and $\rho^{GLO}$ are the correlation between the GPS code and carrier phase observations and the GLONASS code and carrier phase observations, respectively.

The components of $Q_{GPS}^{DD}$ and $Q_{GLO}^{DD}$ matrices ($\sigma_{{\left[i\right]}^{sys}}^{2}$) can be defined as a function of the satellite elevation angle. However, smartphones are equipped with linearly polarized antennas due to the low cost and limited space, resulting in degraded GNSS measurement quality compared with the high-end geodetic GNSS receivers. Therefore, the traditional elevation-dependent weighting method is not appropriate when employing the GNSS observations obtained from Android smartphones. Instead, the C/N0-dependent weighting method is a better option. In this contribution, we will use a combination of C/N0 and elevation-dependent weighting methods. The C/N0/elevation-based stochastic model of GNSS observations ($\sigma_{\left[i\right]}^{2}$) is of the form

(8) $$\sigma_{\left[i\right]}^{2}=\frac{10^{\frac{-{C/N0}_{i}}{10}}}{\sin E_{i}}$$

in which ${C/N0}_{i}$ is the carrier-to-noise ratio value and $E_{i}$ is the satellite elevation angle. In Equations (6) and (7), $a_{type}^{sys}$ is a constant needed to be determined for each device, each constellation (*sys*: GPS and GLONASS), and each observation type (*type*: code and phase). We aim to estimate these unknown values using the LS-VCE method for the Samsung S20 device. We use the following values as the primary values denoted as “nominal stochastic model”:

- For GPS: $a_{code}^{GPS}=10^{5}$, $a_{phase}^{GPS}=10^{3}$ indicating that code observables are 10 times noisier than the carrier phase observables, and $\rho^{GPS}=0$ indicating there is no correlation between the GPS code and phase observables;
- For GLONASS: $a_{type}^{GLO}=2\times a_{type}^{GPS}=2\times 10^{5}$ indicating that GLONASS observables are $\sqrt{2}$ times nosier than the GPS observables and $\rho^{GLO}=0$ indicating there is no correlation between the GLONASS code and carrier phase observables.

We will also estimate these values for the two systems and two observation types as well as possible correlations (i.e., $a_{code}^{GPS}$, $a_{phase}^{GPS}$, $\rho^{GPS}$ for GPS and $a_{code}^{GLO}$, $a_{phase}^{GLO}$, $\rho^{GLO}$ for GLONASS). The unknown vector of the stochastic model is then $\sigma ={\left[\begin{array}{cccccc} a_{code}^{GPS} & a_{phase}^{GPS} & \rho^{GPS} & a_{code}^{GLO} & a_{phase}^{GLO} & \rho^{GLO} \end{array}\right]}^{T}$ and their corresponding cofactor matrices are provided in Table 1. The LS-VCE estimates can then be obtained as $\hat{\sigma }={\left[\begin{array}{cccccc} {\hat{a}}_{code}^{GPS} & {\hat{a}}_{phase}^{GPS} & {\hat{\rho }}^{GPS} & {\hat{a}}_{code}^{GLO} & {\hat{a}}_{phase}^{GLO} & {\hat{\rho }}^{GLO} \end{array}\right]}^{T}=N^{-1}l$.

## 3. PPP Mathematical Model

After estimating the stochastic model using the LS-VEC method, we aim to investigate how introducing a more reliable stochastic model affects the performance of PPP using Android smartphones. PPP is an efficient technique to provide real-time precise positioning using precise satellite orbits and satellite clocks provided, e.g., by the International GNSS Service (IGS). Additional correction terms must also be considered in PPP processing. They include satellite and receiver antenna offsets, carrier phase wind-up, and site displacement effects, including solid Earth tides, ocean tide loading, and polar motion [30]. PPP can also be implemented in smartphone observations. Early smartphones only provided single-frequency and mostly GPS-only observations. In recent years, dual-frequency GNSS smartphones have also been launched. In this contribution, we focus on the noise assessment of GNSS observations from the Samsung S20 device, which is a dual-frequency smartphone supporting L5/E5a frequencies for GPS and Galileo. However, we mainly focus on the single-frequency PPP using the GPS and GLONASS observations as there are no Galileo observations as well as no E1 and E5a frequencies in our collected dataset. This approach can be extended to the dual-frequency multi-GNSS case in the future.

This section consists of two subsections. Section 3.1 provides some explanations about the functional model (SF-PPP) used in the contribution while in Section 3.2, the PPP stochastic model used here is described.

### 3.1. Functional Model

The PPP functional model used in this contribution is the single-frequency uncombined PPP model (SF-PPP) (L1 only). One can further generalize the model to the dual-frequency case (DF-PPP). The undifferenced GNSS code and carrier phase observations for the satellite *s* and the receiver *r* on frequency *j* are as follows [29]:

(9) $$\begin{array}{l} E\left(P_{r,j}^{s}\right)=\rho_{r}^{s}+T_{r}^{s}+{cdt}_{r}-c{dt}^{s}+\gamma_{j}I_{r,1}^{s}+b_{r,j}+b_{,j}^{s}\\ E\left(\Phi_{r,j}^{s}\right)=\rho_{r}^{s}+T_{r}^{s}+{cdt}_{r}-c{dt}^{s}-\gamma_{j}I_{r,1}^{s}+\lambda_{j}N_{r,j}^{s}+B_{r,j}+B_{,j}^{s} \end{array}$$

where $P_{j}$ and $\Phi_{j}$ denote the pseudorange and carrier phase observations on the frequency *j* in meters; ρ is the geometric range between satellite and receiver as a function of the satellite and the receiver coordinates; *T* is the tropospheric delay (m) which can be split into dry and wet parts; c is the vacuum speed of light (m/s); ${dt}_{r}$ and ${dt}^{s}$ are the receiver and satellite clock errors (s), respectively; $I_{r,1}^{s}$ is the first-order slant ionospheric delay on frequency L1 (m); γj=f12fj2 is the frequency-dependent multiplier factor (in the case of L1 frequency $\gamma_{j}=1$); $f_{j}\;$ is the corresponding frequency; $\lambda_{j}$ is the corresponding carrier phase wavelength (m); $N_{r,j}^{s}$ denotes the integer carrier phase ambiguity term in the cycle; $b_{r,j}$ and $B_{r,j}$ denote the frequency-dependent receiver pseudorange and carrier phase hardware delays (biases), respectively; and $b_{,j}^{s}$ and $B_{,j}^{s}$ are the frequency-dependent satellite pseudorange and carrier phase hardware delays (biases), respectively.

The satellite clock errors, which are available from the IGS through the precise clock products, are provided based on the ionosphere-free linear combination of code observations on L1 and L2 frequencies, i.e., P1 and P2 [10]. Employing the satellite clock errors provided by the IGS for the original code and carrier phase observations in the PPP model introduces an additional bias in the observations. To compensate for this bias, one must consider the satellite differential code biases (DCB), which are available from the IGS as well. Let us consider the precise satellite clock errors $c{dt}^{s,IF}$ provided by the IGS as ${cdt}^{s,IF}=c{dt}^{s}-b_{,IF\left(1,2\right)}^{s}$ where $b_{,IF\left(1,2\right)}^{s}=\alpha_{IF}^{1,2}b_{,1}^{s}+\beta_{IF}^{1,2}b_{,2}^{s}$ is the satellite ionosphere-free code bias in which the coefficients αIFi,j=fi2fi2−fj2 and βIFi,j=1−αIFi,j=−fj2fi2−fj2.

The observation minus calculation (OMC) terms of the uncombined code measurement on frequency *j* can be written as follows:

(10) $$E\left(\Delta P_{r,j}^{s}+c{dt}^{s,IF}-\left(b_{,j}^{s}-b_{,IF\left(1,2\right)}^{s}\right)\right)={g_{r}^{s}}^{T}\Delta x_{r}+T_{r}^{s}+{\tilde{cdt}}_{r}+\gamma_{j}I_{r,1}^{s}$$

where ${\tilde{cdt}}_{r}={cdt}_{r}+b_{r,j}$, $b_{,j}^{s}-b_{,IF\left(1,2\right)}^{s}$ is a function of satellite differential code bias ${{DCB}_{12}^{s}=b}_{,1}^{s}-b_{,2}^{s}$ which is known (from the IGS products); $g_{r}^{s}$ is the line-of-sight vector between the satellite and receiver; and $\Delta x_{r}$ is the receiver position increment error. Similarly, for the carrier phase observation equation, we have

(11) $$E\left(\Delta \Phi_{r,j}^{s}+c{dt}^{s,IF}\right)={g_{r}^{s}}^{T}\Delta x_{r}+T_{r}^{s}+{\tilde{cdt}}_{r}-\gamma_{j}I_{r,1}^{s}+\lambda_{j}{\tilde{N}}_{r,j}^{s}$$

where $\lambda_{j}{\tilde{N}}_{r,j}^{s}=\lambda_{j}N_{r,j}^{s}+B_{r,j}-b_{r,j}+B_{,j}^{s}-b_{,IF\left(1,2\right)}^{s}$. In the case of L1-only observations (j=1), the linearized observation equations given in Equations (10) and (11) can be written as follows:

(12) $$\left\{\begin{array}{l} E\left(\Delta P_{r,1}^{s}+c{dt}^{s,IF}+\frac{1}{\gamma_{2}-1}{DCB}_{1,2}^{s}\right)={g_{r}^{s}}^{T}\Delta x_{r}+T_{r}^{s}+{\tilde{cdt}}_{r}+I_{r,1}^{s}\\ E\left(\Delta \Phi_{r,1}^{s}+c{dt}^{s,IF}\right)={g_{r}^{s}}^{T}\Delta x_{r}+T_{r}^{s}+{\tilde{cdt}}_{r}-I_{r,1}^{s}+\lambda_{1}{\tilde{N}}_{r,1}^{s} \end{array}\right.$$

where $\gamma_{j}=1$, ${\tilde{cdt}}_{r}={cdt}_{r}+b_{r,1}$, $\lambda_{1}{\tilde{N}}_{r,1}^{s}=\lambda_{1}N_{r,1}^{s}+B_{r,1}-b_{r,1}+B_{,1}^{s}-b_{,IF\left(1,2\right)}^{s}$. In this equation, $b_{,1}^{s}-b_{,IF\left(1,2\right)}^{s}=\beta_{IF}^{1,2}{DCB}_{12}^{s}=-\frac{1}{\gamma_{2}-1}{DCB}_{1,2}^{s}$ where ${DCB}_{12}^{s}=b_{,1}^{s}-b_{,2}^{s}$ is the satellite DCBs between the first and second frequency bands [31,32].

### 3.2. Stochastic Model

This section outlines the stochastic model of the undifferenced and uncombined GNSS observations. When utilizing the GNSS observations from smartphones, it is more favorable to use the C/N0-dependent weighting method instead of the elevation-dependent weighting approach. In this study, we instead use a combination of both the C/N0 and elevation-dependent weighting models. The C/N0/elevation-based variance of GNSS observations ($\sigma_{type,i}^{2,sys}$) is given as

(13) $$\sigma_{type,i}^{2,sys}=\frac{a_{type}^{sys}10^{\frac{-{C/N0}_{i}}{10}}}{\sin E_{i}}$$

in which sys denotes the constellation type (GPS or GLONASS); type defines the observation type (code or phase); and $\sigma_{type,i}^{2,sys}$ is the variance of observation in m^2^ as a function of its signal-to-noise ratio value ${C/N0}_{i}$ and its elevation angle $E_{i}$. In this formula, $a_{type}^{sys}$ is a constant that has been estimated for each constellation (GPS and GLONASS) and each observation type (code and phase) uses the LS-VCE method explained in the previous section.

Considering GPS and GLONASS observations, the covariance matrix of the undifferenced observations is of the form:

(14) $$Q_{y}={\mathrm{blkdiag}(Q}_{GPS},Q_{GLO})$$

in which $Q_{GPS}$ and $Q_{GLO}$ are the two covariance matrices for the GPS and GLONASS systems, respectively. Assuming $n_{1}$ and $n_{2}$ visible satellites for GPS and GLONASS, respectively, $Q_{GPS}$ and $Q_{GLO}$ can be expressed as follows:

(15) $$Q_{GPS}=\left[\begin{array}{cc} Q_{code}^{GPS} & Q_{code,phase}^{GPS}\\ sym. & Q_{phase}^{GPS} \end{array}\right]\;\mathrm{and}\;Q_{GLO}=\left[\begin{array}{cc} Q_{code}^{GLO} & Q_{code,phase}^{GLO}\\ sym. & Q_{phase}^{GLO} \end{array}\right]$$

where $Q_{code}^{GPS}$ and $Q_{phase}^{GPS}$ are the two $n_{1}\times n_{1}$ matrices related to the GPS code and carrier phase observations, $Q_{code}^{GLO}$ and $Q_{phase}^{GLO}$ are the two $n_{2}\times n_{2}$ matrices related to the GLONASS code and carrier phase observations and sym is the notation of a symmetric matrix. $Q_{type}^{sys}$ for each system and each observation type can also be obtained as follows:

(16) $$Q_{type}^{sys}=diag\left(\left[\sigma_{type,1}^{2,sys},\sigma_{type,2}^{2,sys},\ldots ,\sigma_{type,n_{i}}^{2,sys}\right]\right)$$

where diag is the diagonal operator, sys could be “GPS” or “GLO”, type could be “code” or “phase” and $n_{i}$; *i* = 1, 2 is the number of visible satellites for GPS and GLO, respectively. In Equation (15), $Q_{code,phase}^{GPS}$ is a matrix showing the possible correlation among the code and phase observations of each constellation. It is of the form:

(17) $$Q_{code,phase}^{sys}=diag\left(\rho^{sys}\left[\sigma_{code,1}^{sys}\sigma_{phase,1}^{sys},\sigma_{code,2}^{sys}\sigma_{phase,2}^{sys},\ldots ,\sigma_{code,n_{i}}^{sys}\sigma_{phase,n_{i}}^{sys}\right]\right)$$

where $\rho^{sys}$ is the possible correlation among the code and phase observations estimated by using the LS-VCE method.

## 4. Experimental Results

In this section, the noise characteristics of GPS, the GLONASS code, and carrier phase observations from the two Samsung S20 devices are first investigated. The positioning performance of the SF-PPP model while employing the reliable stochastic model is then evaluated.

### 4.1. Noise Assessment of Samsung S20

In this section, two Samsung S20 devices, namely Samsung S20 Black (S20B) and Samsung S20 White (S20W), were placed close to each other on a pillar with the true coordinates making a very short baseline (about 5 cm). The data were collected on 4 February 2023 for 3 h from 00:30:00 to 03:30:00 UTC with a sampling interval of 1 s (10,800 epochs).

**Figure 1:**
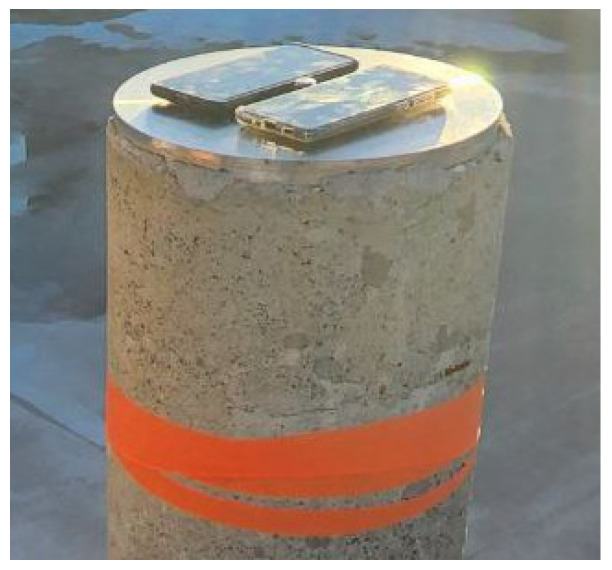
Two Samsung S20 smartphones generate a very short baseline.

Figure 1 shows the location of the two Samsung S20 smartphones which are on top of a geodetic pillar with known coordinates on the rooftop of the civil engineering building (pillar N2), University of Calgary, Calgary, Canada. Table 2 provides the coordinates of pillar N2 and Table 3 gives a brief summary of the experiment.

**Figure 2:**
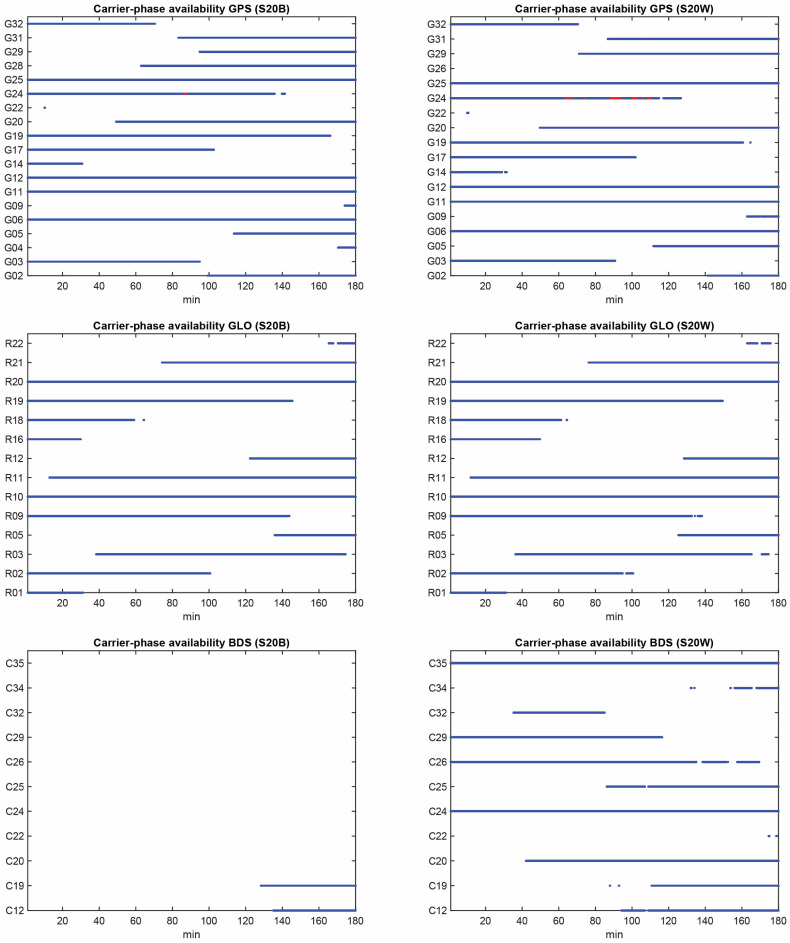
GPS, GLONASS, and BeiDou carrier phase continuity for S20B and S20W.

The Samsung S20 is a smartphone that supports both L5 and E5a frequencies for GPS and Galileo, respectively. However, the Galileo observations, including E1 and E5a frequencies, were not logged in the collected dataset for some unknown reason. Furthermore, there were only a few GPS satellites with L5 signals observed throughout the entire data collection period. Hence, our attention will be directed toward the single-frequency GPS and GLONASS observations. As the proposed smartphone stochastic modeling approach relies on employing the DD observations, we need to first analyze the availability and continuity of GNSS observations from both devices, as well as the common satellites between the two devices. Figure 2 provides the GNSS carrier phase continuity on the L1 frequency for the two devices. In this figure, the red dots denote the epochs in which the carrier phase observations were missing while the code observations were still observed. This figure indicates that the carrier phase continuity is different for the two Samsung S20 devices.

**Figure 3:**
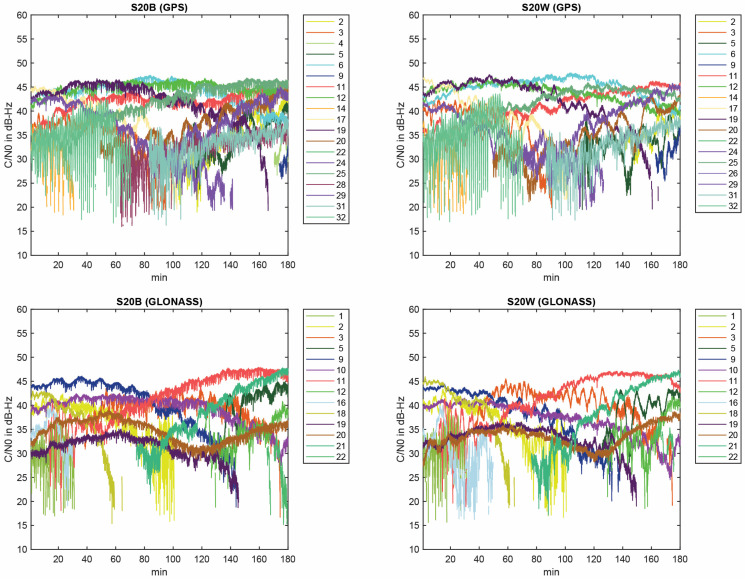
C/N0 values for GPS and GLONASS on the first frequency for both devices.

**Figure 4:**
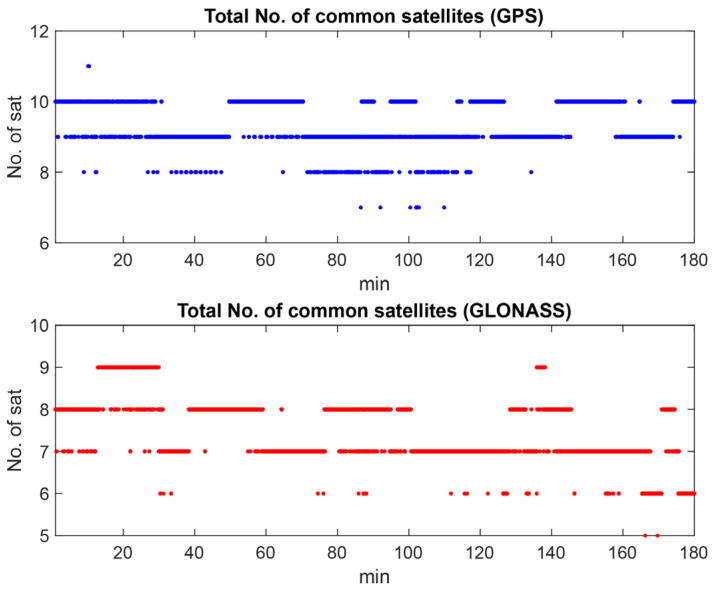
Total number of common GPS and GLONASS satellites between S20B and S20A on L1 frequency.

**Figure 5:**
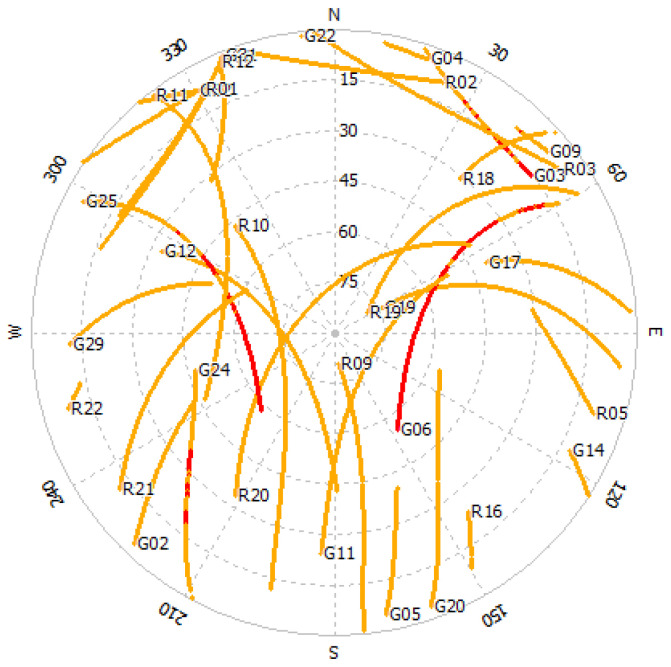
Sky plot for Samsung S20B (red: GPS satellites with L5 signals observed).

According to the figures, GPS and GLONASS are common constellations between two devices with an adequate number of tracked satellites, while the BeiDou does not provide enough observations in the S20B dataset. To continue, we will therefore focus on the noise assessment of GPS and GLONASS observations on the L1 frequency. Shown in Figure 3 are the C/N0 values for GPS and GLONASS on the first frequency for both devices. The plot reveals that the performance of the two Samsung S20 devices is almost similar in terms of the recorded C/N0 values. Figure 4 also illustrates the total number of common GPS/GLONASS satellites between Samsung S20 Black and Samsung S20 White on the L1 frequency. The geometry of GPS and GLONASS satellites in view is also presented in the Sky plot in Figure 5.

**Figure 6:**
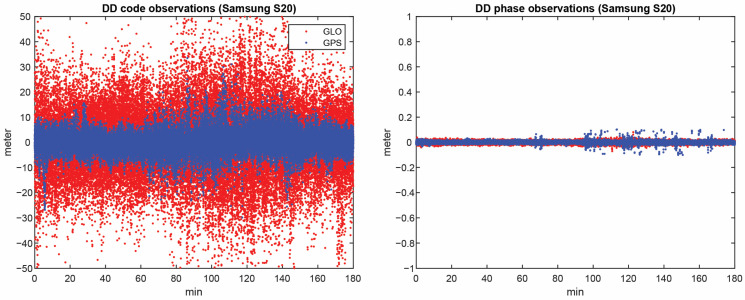
DD observations of GPS and GLONASS for all visible satellites: (**left**) code and (**right**) phase observations.

Following the assessment of the consistency of the C/N0 and the number of common satellites between the devices, we obtained the DD code and carrier phase observations for GPS and GLONASS. PRN 06 and PRN 20 were selected as the reference satellites for GPS and GLONASS, respectively. The DD code and phase observations for all visible satellites on the L1 frequency for both constellations are presented in Figure 6. Table 4 provides the mean and RMS of the DD code observations for GPS and GLONASS on the L1 frequency. Based on the results, the quality of the GLONASS code observations on the L1 frequency is worse than the other two systems.

**Figure 7:**
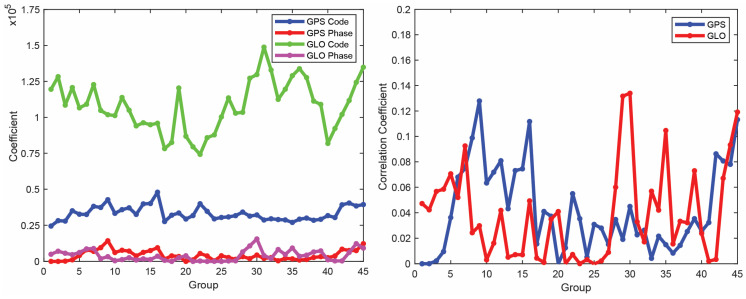
Groupwise estimate of coefficient ($a_{type}^{sys}$; sys: GPS and GLONASS and type: code and phase) on L1 frequency as well as correlations among code and phase observations of each constellation (i.e., $\rho^{GPS}$ and $\rho^{GLO}$ ).

We now employ the LS-VCE method on the DD code and phase observations on the L1 frequency. First, the entire time span of the smartphone observations (10,800 epochs) is divided into 45 groups, each consisting of 240 consecutive epochs (4 min). The unknown (co)variance parameters $\sigma ={\left[\begin{array}{cccccc} a_{code}^{GPS} & a_{phase}^{GPS} & \rho^{GPS} & a_{code}^{GLO} & a_{phase}^{GLO} & \rho^{GLO} \end{array}\right]}^{T}$ can then be separately estimated for each group. The final solution will be the average over all groups. Figure 7 shows the groupwise estimates of $a_{type}^{sys}$ for the code/phase observations of GPS and GLONASS on the first frequency as well as estimated correlations among the code and phase observations of each constellation (i.e., $\rho^{GPS}$ and $\rho^{GLO}$). The final estimates are just the arithmetic mean of the individual estimates over the 45 groups. Table 5 presents the mean value of the estimated coefficients ($a_{type}^{sys}$ where sys refers to GPS and GLONASS and type denotes the observation type, i.e., code and phase) on L1 frequency as well as the correlation between code and carrier phase observations. The results indicate that there is no significant correlation between the code and carrier phase observations of GPS and GLONASS.

**Figure 8:**
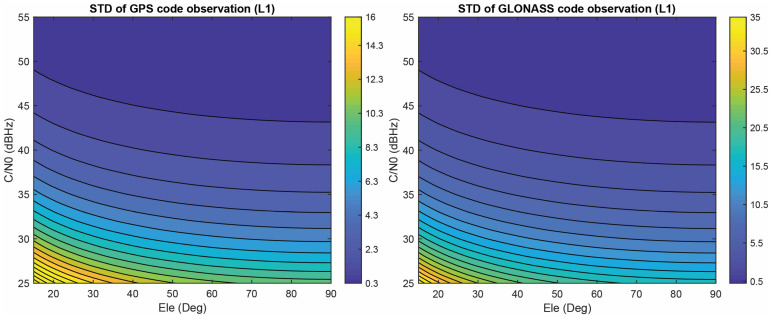
Estimated STD for GPS and GLONASS code observations on L1 frequency (in meters).

Considering ${\hat{a}}_{code}^{GPS}=0.35\times 10^{5}$ and ${\hat{a}}_{code}^{GLO}=1.08\times 10^{5}$, the standard deviation (STD) of the code observations for GPS and GLONASS on the first frequency can be observed in Figure 8 as a function of the C/N0 value and elevation angle.

Yong et al. [34] also investigated the noise characteristics of the Samsung S20 and Google Pixel 4 smartphones by fitting empirical 95% confidence ellipses/levels to the formal counterparts, as derived from the corresponding variance-covariance (VCV) matrices of the positions. The empirical VCV-matrix was obtained by comparing estimated positions to precise benchmark coordinates, while the formal VCV-matrix was obtained from the means of all single-epoch formal VCV-matrices over the observation span. The study obtained the undifferenced and zenith-referenced STDs in three different setup configurations: zero-baseline external antennas, short-baseline external antennas, and short-baseline internal antennas. The authors assumed the same code and phase STDs for all frequencies (L1, L5, E1, E5a, and B1) and constellations. In the case of the Samsung S20 with short-baseline internal antennas, the corresponding STD values for code and carrier phase observations were 0.003 m and 2.327 m, respectively. In the current research, we utilized the LS-VCE method, which allowed us to estimate frequency/observation type/system-dependent precision (i.e., code vs. carrier phase, L1 vs. L5, and GPS vs. other constellations) as well as any covariances between different observation types (e.g., between code and carrier phase). However, it is important to note some limitations associated with the proposed method. First, the proposed method relies on constructing the DD observations between two smartphones, which necessitates a sufficient number of common satellites between the devices. However, due to the low-cost linearly polarized antennas used in smartphones, they may not be able to track all the same satellites. Second, implementing the LS-VCE method can be time-consuming for two reasons: (1) it is an iterative method and (2) creating matrices with large sizes may surpass the maximum array size allowed. Third, the dataset used in the contribution did not include the measurements on the L5 frequency, thereby limiting the ability to evaluate the noise of observations on the L5 frequency.

### 4.2. SF-PPP Performance Employing Reliable Stochastic Model

**Figure 9:**
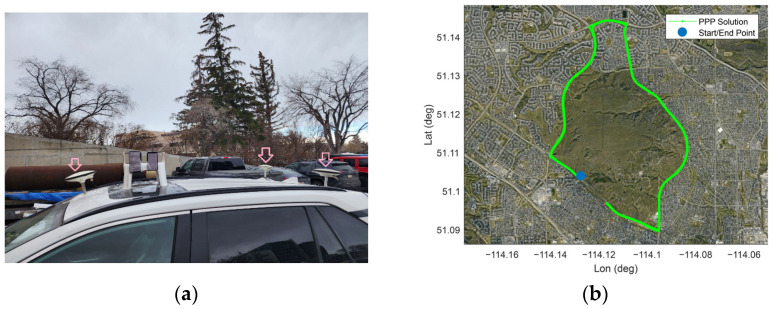
A kinematic experiment conducted on 22 April 2022: (**a**) test configuration and (**b**) reference trajectory [33].

The performance of the SF-PPP model when using the reliable stochastic model is investigated in kinematic mode using a dataset from the same dual-frequency Samsung S20 device as the previous Section 4.1. A kinematic test was carried out on 22 November 2022 with a duration of nearly 1 h in a mostly open-sky environment with overpasses, in Calgary, Alberta, Canada. Figure 9 displays the kinematic test configuration and the reference vehicle’s path in this experiment.

For the kinematic experiment, we used three geodetic receivers, two U-blox F9Ps, and one Septentrio AsteRx-m2, as indicated by the three pick arrows in Figure 9. Additionally, we placed six smartphones on the vehicle roof to be used for future research. However, for this particular study, we only utilized the dataset from the Samsung S20 Black. The reference trajectory of the vehicle during the kinematic experiment was obtained by the RTK fixed solutions from the three geodetic receivers (shown by the three pick arrows in Figure 9) as the rover receivers and a geodetic receiver on a geodetic pillar (with true position) on the rooftop of the civil engineering building at the University of Calgary as the base receiver. The offsets between all units were measured and applied prior to comparison. Table 6 provides GNSS data information and processing setting.

Considering ${\hat{a}}_{code}^{GPS}=0.35\times 10^{5}$, ${\hat{a}}_{code}^{GPS}=0.04\times 10^{5}$ for GPS and ${\hat{a}}_{code}^{GLO}=1.08\times 10^{5}$, ${\hat{a}}_{code}^{GLO}=0.04\times 10^{5}$ for GLONASS as reported in Table 5, one can obtain the C/N0/elevation-based stochastic model of GNSS observations ($\sigma_{\left[i\right],type}^{2,sys}$) as follows:

(18) $$\sigma_{\left[i\right],type}^{2,sys}={\hat{a}}_{type}^{sys}\frac{10^{\frac{-{C/N0}_{i}}{10}}}{\sin E_{i}}$$

**Figure 10:**
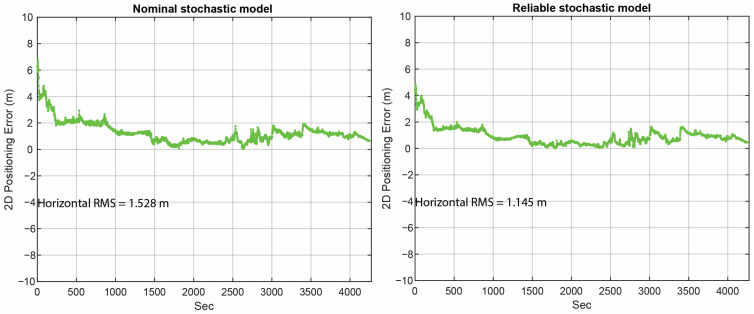
Horizontal positioning error in two cases: (**left**) nominal stochastic model and (**right**) reliable stochastic model.

**Figure 11:**
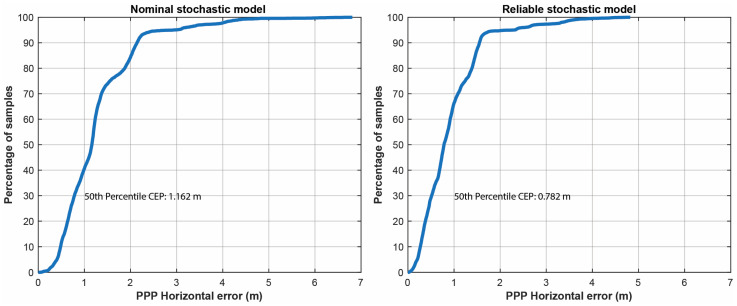
Cumulative distribution error plot of horizontal positioning error: (**left**) nominal stochastic model and (**right**) reliable stochastic model.

The horizontal positioning error in the cases of the SF-PPP model using the nominal stochastic model (i.e., $a_{code}^{GPS}=10^{5}$, $a_{phase}^{GPS}=10^{3}$ and $a_{type}^{GLO}=2{\times a}_{type}^{GPS}={2\times 10}^{5}$) and the reliable stochastic model (considering the estimated scale factors ${\hat{a}}_{type}^{sys}$) are provided in Figure 10. Please note that the values displayed in Figure 10 were calculated using all positioning solutions, including the convergence period. Figure 11 depicts the cumulative distribution error plot of the horizontal positioning error for both cases. The results indicate that incorporating a more reliable stochastic model resulted in an improvement in positioning performance, specifically a 25.1% decrease in horizontal root mean square (RMS) and a 32.7% decrease in the 50th percentile error. Furthermore, the maximum error decreased from approximately 7 m to about 5 m when the reliable stochastic model was introduced. These findings demonstrate the positive impact of employing a reliable stochastic model in this kinematic experiment.

Several studies have focused on investigating the performance of PPP smartphone positioning using different weighting models. Some examples include [19,33,35,36,37,38]. Li et al. [19] utilized a weighting approach that combined the elevation angle and C/N0 for smartphone-based GNSS PPP. The proposed method was then assessed through GNSS kinematic experiments using the Xiaomi MI8 device. The RMS values of the PPP errors in the east, north, and up components were 1.00 m, 0.62 m, and 2.22 m, respectively, when utilizing the combined weighting scenario. This represents an improvement compared to using either the elevation-angle-only weighting or the C/N0-only weighting scenarios. Zangenehnejad et al. developed their own CSV to RINEX converter in [33] and applied PPP to GNSS observations collected from a Xiaomi Mi8 smartphone based on a C/N0-dependent stochastic model. They found that the 50th percentile horizontal error was 0.330 m in a kinematic test conducted in an open-sky environment. In [35], Liu et al. used GNSS observations from the Xiaomi Mi8 and Samsung S20 smartphones and achieved sub-meter PPP accuracy at the 50th percentile horizontal error. However, no details were provided regarding the employed stochastic model. Wu et al. [36] used dual-frequency GPS (L1/L5) and Galileo (E1/E5a) observations from a Xiaomi Mi8 smartphone. By utilizing an elevation-dependent stochastic model, they demonstrated that the PPP algorithm’s positioning accuracy was at the meter level in kinematic mode, which is comparable to the accuracy of our solution. Chen et al. [37] proposed a modified single-frequency PPP algorithm that estimates separate clock biases for pseudorange and carrier phase observations. They used a Xiaomi Mi8 smartphone and found that the average horizontal and vertical RMS errors based on an elevation-dependent stochastic model were 0.81 m and 1.65 m, respectively. Zhu et al. [38] also utilized a Huawei Mate 30 smartphone to carry out PPP testing in a kinematic mode, employing the C/N0-dependent stochastic model. They could achieve a positioning accuracy of 0.93 m, 0.62 m, and 2.17 m in the east, north, and up directions, respectively. The findings showed that the accuracy was improved by approximately 26.2%, 20.5%, and 20.4% in comparison to the elevation-dependent stochastic model results. Although differences in the measurement environment and employed mathematical models could explain variations in the obtained accuracy, the overall performance of PPP remains consistent across different studies.

## 5. Summary and Conclusions

Thanks to the new API implemented on Android 7 and later versions, the utilization of smartphones for various applications such as cadastral surveying, mapping surveying applications, and navigation has been significantly increasing due to cost-effective GNSS smartphones. However, there are still various challenges that prevent users from obtaining highly accurate results using smartphone observations.

In this study, we have investigated reliable stochastic modeling of smartphone GNSS observations and developed a C/N0 and elevation-dependent weighting method. We obtained a reliable stochastic model using the LS-VCE method and employing the DD code and phase observations from a very short baseline (two Samsung S20 devices placed close to each other on a pillar). The model performance has been evaluated by single-frequency PPP with a Samsung S20 smartphone. The following conclusions can be drawn from our study on reliable stochastic modeling using LS-VCE and application to Samsung S20 smartphones:

1. The estimated coefficients for each constellation and each observation type are as follows: ${\hat{a}}_{code}^{GPS}=0.35\times 10^{5}$, ${\hat{a}}_{code}^{GPS}=0.04\times 10^{5}$ for GPS and ${\hat{a}}_{code}^{GLO}=1.08\times 10^{5}$, ${\hat{a}}_{code}^{GLO}=0.04\times 10^{5}$ for GLONASS as reported in Table 5;
2. There is no significant correlation between the GPS code and carrier phase observations on the L1 frequency. The same conclusions hold for the GLONASS constellation;
3. The results of the study confirmed an improvement of 25.1% and 32.7% on the RMS of horizontal positioning and the 50th percentile error, respectively, when employing the obtained stochastic model. This research provides insights into the potential of high-precision smartphone positioning and highlights the importance of the proper choice of functional and stochastic models in achieving this goal.

Our future research will focus on developing a more reliable stochastic model for other constellations as well as the second frequency (L5/E5a).

## Figures and Tables

**Table 1:** Six cofactor matrices related to six LS-VCE unknowns.

$Q_{1}=blkdiag(\left[\begin{array}{cc} 1 & 0\\ 0 & 0 \end{array}\right]⊗2\left[\begin{array}{cccc} \sigma_{{\left[1\right]}^{G}}^{2}+\sigma_{{\left[2\right]}^{G}}^{2} & \sigma_{{\left[1\right]}^{G}}^{2} & \cdots & \sigma_{{\left[1\right]}^{G}}^{2}\\ \sigma_{{\left[1\right]}^{G}}^{2} & \sigma_{{\left[1\right]}^{G}}^{2}+\sigma_{{\left[3\right]}^{G}}^{2} & \cdots & \sigma_{{\left[1\right]}^{G}}^{2}\\ \vdots & \vdots & \ddots & \vdots \\ \sigma_{{\left[1\right]}^{G}}^{2} & \sigma_{{\left[1\right]}^{G}}^{2} & \cdots & \sigma_{{\left[1\right]}^{G}}^{2}+\sigma_{{\left[n_{1}\right]}^{G}}^{2} \end{array}\right],0_{n_{2}\times n_{2}})$
$Q_{2}=blkdiag(\left[\begin{array}{cc} 0 & 0\\ 0 & 1 \end{array}\right]⊗2\left[\begin{array}{cccc} \sigma_{{\left[1\right]}^{G}}^{2}+\sigma_{{\left[2\right]}^{G}}^{2} & \sigma_{{\left[1\right]}^{G}}^{2} & \cdots & \sigma_{{\left[1\right]}^{G}}^{2}\\ \sigma_{{\left[1\right]}^{G}}^{2} & \sigma_{{\left[1\right]}^{G}}^{2}+\sigma_{{\left[3\right]}^{G}}^{2} & \cdots & \sigma_{{\left[1\right]}^{G}}^{2}\\ \vdots & \vdots & \ddots & \vdots \\ \sigma_{{\left[1\right]}^{G}}^{2} & \sigma_{{\left[1\right]}^{G}}^{2} & \cdots & \sigma_{{\left[1\right]}^{G}}^{2}+\sigma_{{\left[n_{1}\right]}^{G}}^{2} \end{array}\right],0_{n_{2}\times n_{2}})$
$Q_{3}=blkdiag(\left[\begin{array}{cc} 0 & 1\\ 1 & 0 \end{array}\right]⊗2\left[\begin{array}{cccc} \sigma_{{\left[1\right]}^{G}}^{2}+\sigma_{{\left[2\right]}^{G}}^{2} & \sigma_{{\left[1\right]}^{G}}^{2} & \cdots & \sigma_{{\left[1\right]}^{G}}^{2}\\ \sigma_{{\left[1\right]}^{G}}^{2} & \sigma_{{\left[1\right]}^{G}}^{2}+\sigma_{{\left[3\right]}^{G}}^{2} & \cdots & \sigma_{{\left[1\right]}^{G}}^{2}\\ \vdots & \vdots & \ddots & \vdots \\ \sigma_{{\left[1\right]}^{G}}^{2} & \sigma_{{\left[1\right]}^{G}}^{2} & \cdots & \sigma_{{\left[1\right]}^{G}}^{2}+\sigma_{{\left[n_{1}\right]}^{G}}^{2} \end{array}\right],0_{n_{2}\times n_{2}})$
$Q_{4}=blkdiag(0_{n_{1}\times n_{1}},\left[\begin{array}{cc} 1 & 0\\ 0 & 0 \end{array}\right]⊗2\left[\begin{array}{cccc} \sigma_{{\left[1\right]}^{R}}^{2}+\sigma_{{\left[2\right]}^{R}}^{2} & \sigma_{{\left[1\right]}^{R}}^{2} & \cdots & \sigma_{{\left[1\right]}^{R}}^{2}\\ \sigma_{{\left[1\right]}^{R}}^{2} & \sigma_{{\left[1\right]}^{R}}^{2}+\sigma_{{\left[3\right]}^{R}}^{2} & \cdots & \sigma_{{\left[1\right]}^{R}}^{2}\\ \vdots & \vdots & \ddots & \vdots \\ \sigma_{{\left[1\right]}^{R}}^{2} & \sigma_{{\left[1\right]}^{R}}^{2} & \cdots & \sigma_{{\left[1\right]}^{R}}^{2}+\sigma_{{\left[n_{2}\right]}^{G}}^{2} \end{array}\right])$
$Q_{5}=blkdiag(0_{n_{1}\times n_{1}},\left[\begin{array}{cc} 0 & 0\\ 0 & 1 \end{array}\right]⊗2\left[\begin{array}{cccc} \sigma_{{\left[1\right]}^{R}}^{2}+\sigma_{{\left[2\right]}^{R}}^{2} & \sigma_{{\left[1\right]}^{R}}^{2} & \cdots & \sigma_{{\left[1\right]}^{R}}^{2}\\ \sigma_{{\left[1\right]}^{R}}^{2} & \sigma_{{\left[1\right]}^{R}}^{2}+\sigma_{{\left[3\right]}^{R}}^{2} & \cdots & \sigma_{{\left[1\right]}^{R}}^{2}\\ \vdots & \vdots & \ddots & \vdots \\ \sigma_{{\left[1\right]}^{R}}^{2} & \sigma_{{\left[1\right]}^{R}}^{2} & \cdots & \sigma_{{\left[1\right]}^{R}}^{2}+\sigma_{{\left[n_{2}\right]}^{G}}^{2} \end{array}\right])$
$Q_{6}=blkdiag(0_{n_{1}\times n_{1}},\left[\begin{array}{cc} 0 & 1\\ 1 & 0 \end{array}\right]⊗2\left[\begin{array}{cccc} \sigma_{{\left[1\right]}^{R}}^{2}+\sigma_{{\left[2\right]}^{R}}^{2} & \sigma_{{\left[1\right]}^{R}}^{2} & \cdots & \sigma_{{\left[1\right]}^{R}}^{2}\\ \sigma_{{\left[1\right]}^{R}}^{2} & \sigma_{{\left[1\right]}^{R}}^{2}+\sigma_{{\left[3\right]}^{R}}^{2} & \cdots & \sigma_{{\left[1\right]}^{R}}^{2}\\ \vdots & \vdots & \ddots & \vdots \\ \sigma_{{\left[1\right]}^{R}}^{2} & \sigma_{{\left[1\right]}^{R}}^{2} & \cdots & \sigma_{{\left[1\right]}^{R}}^{2}+\sigma_{{\left[n_{2}\right]}^{G}}^{2} \end{array}\right])$

**Table 2:** True coordinate of pillar N2.

Pillar	N2
Y (m)	−1,641,898.085
Y (m)	−3,664,875.558
Z (m)	4,939,969.351
Lat. (Deg.)	51.07942671
Lon. (Deg.)	−114.13282014
H (m)	1116.589

**Table 3:** GNSS data information.

**Device**	Samsung S20 Black (S20B) and Samsung S20 White (S20W)
**Constellations**	GPS (Yes), GLONASS (Yes), Galileo (No observation recorded), BeiDou (Yes), and QZSS (Yes)
**Mode**	Static
**App logger**	GnssLogger (v3.0.5.6)
**RINEX converter**	UofC CSV2RINEX tool [33]
**Date**	4 February 2023
**Duration**	3 h
**Sampling interval**	1 s

**Table 4:** Mean and RMS of the DD code and phase observations on L1 frequency (in meters).

Observation Type	Parameter	GPS	GLONASS
Code	Mean	0.126	0.001
	RMS	3.408	13.596
Phase	Mean	0.001	0.001
	RMS	0.01	0.009

**Table 5:** The mean value of the estimated coefficients ($a_{type}^{sys}$; sys: GPS and GLONASS and type: code and phase) on L1 frequency as well as the correlation among code and phase observations.

Constellation	${\hat{a}}_{code}^{sys}$	${\hat{a}}_{phase}^{sys}$	$\hat{\rho }$
GPS	0.35 × 10^5^	0.04 × 10^5^	0.04
GLONASS	1.08 × 10^5^	0.04 × 10^5^	0.03

**Table 6:** GNSS data information and processing setting.

**Device**	Samsung S20 Black
**App logger**	GnssLogger (v3.0.5.6)
**RINEX converter**	UofC CSV2RINEX tool [32]
**Measurements used**	GPS (L1), GLONASS (L1)
**Mode**	Kinematic
**Date**	22 November 2022
**Duration**	1 h
**Sampling interval**	1 s
**Troposphere model**	Saastamoinen model
**Ionosphere model**	Global ionospheric maps (GIM)
**Functional model**	SF PPP model
**Stochastic model**	C/N0 and elevation weighting function (nominal and estimated stochastic model)
**Elevation mask angle**	10 deg
**C/N0 mask**	20 dB-Hz
**Satellite orbit**	CODE MGEX precise ephemerides (5 min interval)
**Clock error**	CODE MGEX precise clock (1 s interval)
**Satellite DCB correction**	CAS DCBs in Bias SINEX (BSX) format

## Data Availability

The data that support the findings of this study are available upon request from the corresponding author, via farzaneh.zangenehnej@ucalgary.ca.
